# The Geographical Distribution of Leadership in Globalized Clinical Trials

**DOI:** 10.1371/journal.pone.0045984

**Published:** 2012-10-10

**Authors:** Jarno Hoekman, Koen Frenken, Dick de Zeeuw, Hiddo Lambers Heerspink

**Affiliations:** 1 Department of Clinical Pharmacology, University of Groningen, University Medical Center Groningen, Groningen, The Netherlands; 2 School of Innovation Sciences, Eindhoven University of Technology, Eindhoven, The Netherlands; Cincinnati Childrens Hospital Medical Center, United States of America

## Abstract

**Background:**

Pharmaceutical trials are mainly initiated by sponsors and investigators in the United States, Western Europe and Japan. However, more and more patients are enrolled in Central and Eastern Europe, Latin America and Asia. The involvement of patients in new geographical settings raises questions about scientific and ethical integrity, especially when experience with those settings is lacking at the level of trial management. We therefore studied to what extent the geographical shift in patient enrolment is anticipated in the composition of trial management teams using the author nationalities on the primary outcome publication as an indicator of leadership.

**Methods and Findings:**

We conducted a cohort-study among 1,445 registered trials in www.clinicaltrials.gov that could be matched with a primary outcome publication using clinical trial registry numbers listed in publications. The name of the sponsor and the enrolment countries were extracted from all registrations. The author-addresses of all authors were extracted from the publications. We searched the author-address of all publications to determine whether enrolment countries and sponsors listed on registrations also appeared on a matched publication. Of all sponsors, 80.1% were listed with an author-address on the publication. Of all enrolment countries, 50.3% appeared with an author-address on the publication. The listing of enrolment countries was especially low for industry-funded trials (39.9%) as compared to government (90.4%) and not-for-profit funding (93.7%). We found that listing of enrolment countries in industry-funded trials was higher for traditional research locations such as the United States (98.2%) and Japan (72.0%) as compared to nontraditional research locations such as Poland (27.3%) and Mexico (14.1%).

**Conclusions:**

Despite patient enrolment efforts, the involvement of researchers from nontraditional locations in trial management as measured by their contribution to manuscript writing is modest. This division of labor has significant implications for the scientific and ethical integrity of global clinical research.

## Introduction

Collaboration and globalization are defining characteristics of contemporary scientific knowledge production, with the randomized clinical trial being a textbook-like example. The conduct of clinical trials necessitates the collaborative involvement of many researchers with roles ranging from designing protocols and enrolling human subjects for data collection to analyzing data and preparing manuscripts for publication. Standardization and harmonization of these research practices - as envisaged in the ICH-GCP guideline – has made the travelling of clinical data between geographically dispersed research sites less complicated [1]. This has facilitated globalization of clinical trials with increasing involvement of researchers from nontraditional research locations, especially in Central and Eastern Europe, Latin America and Asia [2], [3], [4].

However, as clinical trials become ever more global, worries have been voiced over the division of roles and responsibilities in those projects. Critics argue that global clinical trials are primarily conducted for the benefit of a small group of leading scientists and companies located in the major pharmaceutical markets. Investigators from nontraditional research locations are only hired in these projects to bring in their patients as ‘experimental subjects’, without having significant roles in defining research questions, analyzing the data or drafting manuscripts for publication [1], [5], [6].

These concerns might be particularly warranted in large scale multi-center clinical trials that require the appointment of trial management and evaluation teams such as Executive Committees, Steering Committees, Data Safety Monitoring Boards and Outcome Adjudication Committees. These bodies take overall responsibility for the integrity of the study and the knowledge production process and they also have a major stake in writing the final clinical trial reports for publication.

The constitution of trial management often takes place in consultation between the sponsor and the first appointed principal investigator. In principle, membership can be geographically decoupled from more operational tasks executed at clinical sites. The quality and integrity of clinical trials may however be compromised when knowledge of the actual clinical research situation on the ground is lacking at the level of scientific management. Geographical differences in the patho-physiology and behavior of patients may hinder the execution of complex research protocols, and introduce geographical differences in drug adherence, drug response, side-effect profiles and ultimately clinical trial outcomes. At the same time, the integrity of individual trials may be at stake in the absence of close oversight, especially when local regulators and health care professionals are less experienced with clinical trial conduct. In order to enhance the scientific and ethical integrity of clinical trials, it is thus recommended that trial management reflects the geographical diversity of the studied patient populations.

Since there is however no large-scale quantitative data available on the relation between trial participation and appointment in trial management, we set out to quantify the extent of this phenomenon. More specifically, we study the extent to which authors on primary outcome publications – as an indicator of trial leadership - are located in the countries where clinical researchers are involved with human subjects at clinical sites.

## Methods

### 1. Data collection

**Figure 1:**
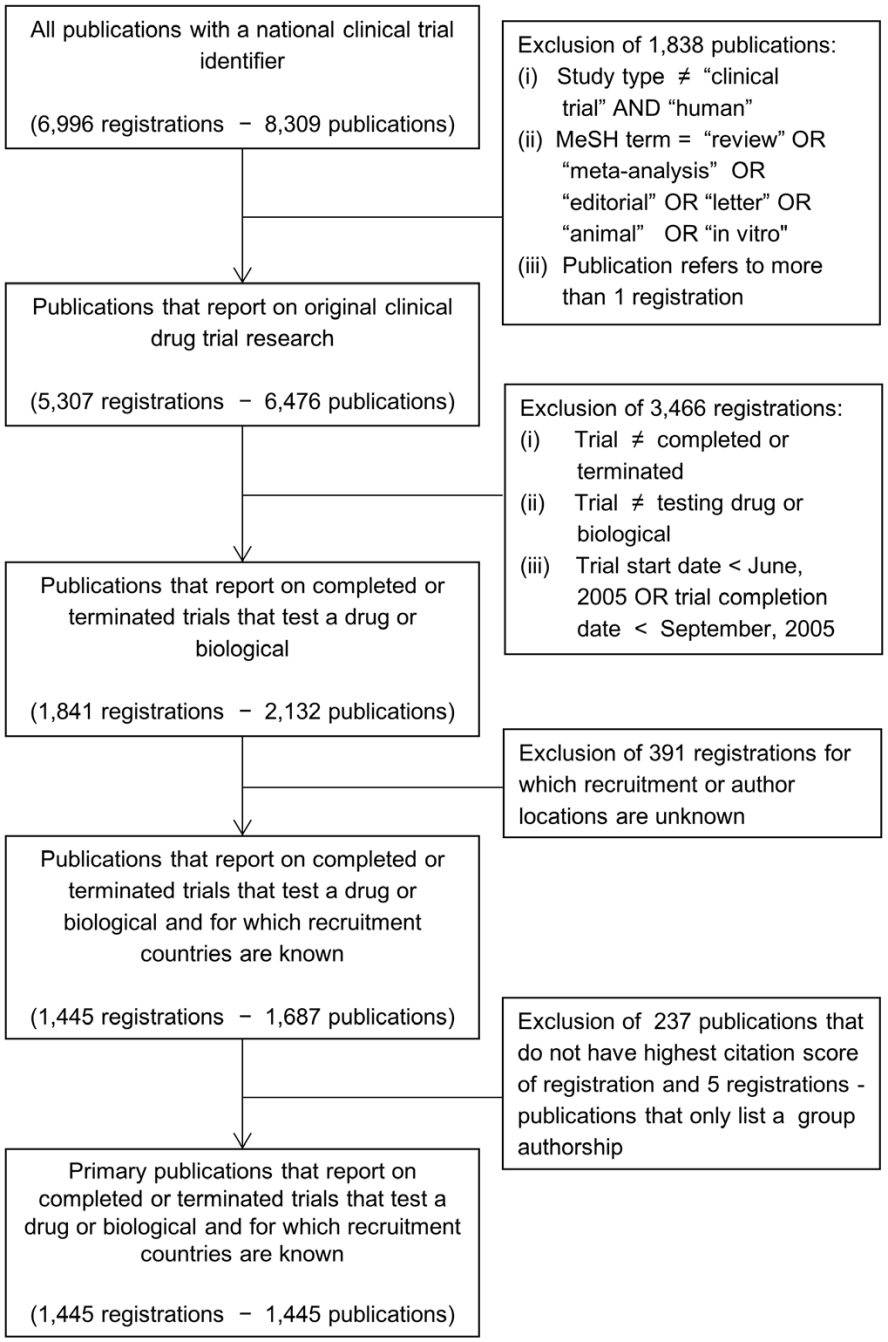
Inclusion flowchart of sample.

Our cohort consists of publications describing the primary results of clinical trials that are registered on the website www.clinicaltrials.gov (Figure 1). To establish the link between registrations and publications, we searched MEDLINE via PUBMED to find all publications that list a registry number of a clinical trial (national clinical trial identifier) in the title, abstract or as a secondary source ID. In order to obtain a sample that only contains original clinical trial publications, the MEDLINE search was limited by study type (“clinical trial" and “human") and excluded publications with the following study types or MeSH terms: “editorials", “letters", “in vitro", “animal", “review", “meta-analysis". We also excluded publications if they made reference to more than one clinical trial identifier.

Using the trial registry number, the publication sample was matched with all completed or terminated trials that were registered in www.clinicaltrials.gov as of January 2011 and set out to test a drug or a biological. We only focused on trials that had either a start date after June, 2005 or a completion date after September, 2005, by which trial registration was mandated by the International Committee of Medical Journal Editors before onset [6].

Our prime interest was to determine whether sponsors and enrolment countries listed on clinical trial registrations were also listed as authors on a matched outcome publication. To obtain the sponsors listed on clinical trial registrations we extracted the sponsor names mentioned in the lead sponsor field of www.clinicaltrials.gov. To obtain the enrolment countries, we extracted for every registration all unique countries listed in the study location field of www.clinicaltrials.gov. Because information in the study location field was not always reported, we had to exclude 391 protocols. The search matched 1,450 registrations to 1,687 publications (Figure 1).

143 of the 1,450 registrations had a match with more than one clinical trial publication. As we were interested in the primary publication following clinical trial conduct, we retrieved the citation impact scores of all publications from the citation databank Scopus Elsevier and determined for each registration which publication received the highest number of citations. We assumed here that the publication that received the highest number of citations was the primary outcome publication. In case two publications received an equal amount of citations, we took the earliest publication. In addition, we removed an additional 5 publications because they only listed a group-authorship in the byline of the article.

For the remaining 1,445 publications, all author names were extracted from both PUBMED and Scopus Elsevier and the number of authors listed on each publication was compared. In 82 publications there was disagreement between PUBMED and Scopus Elsevier on the number of authors. This disagreement was resolved by manually checking the full-text of the article.

We subsequently retrieved the affiliation and country of origin of all authors that were listed in the address field of the publications. We downloaded this information from Scopus Elsevier, which systematically keeps track of address information of authors. For 226 publications, affiliation or address information of at least one of the authors was missing. In these cases we manually retrieved the data from the full-text of the publication. We were successful in doing this for all but four authors. After retrieving address information, we listed per publication the name, organization and country of all 14,298 authors.

### 2. Analysis

Registrations and publications were 1∶1 matched in 1,445 registration-publication pairs. For all registration-publication pairs we determined whether the enrolment countries on the registration were mentioned as a country in an author-address on a matched publication. Based on this information we computed authorship rates per country which were defined as the percentage of registered enrolment efforts per country that resulted in an author-address of that country on a matched publication. Evidently, the maximum authorship rate of a country is 100 percent, which would indicate that every time a patient from a particular country is enrolled in a clinical trial, the country is also represented by an author on a matched publication. We computed authorship rates for all registrations and broken down by funding types (i.e. industry, government, other not for profit).

We also determined whether the lead sponsor on the registration was listed with an author-address on the matched publication. Next to exact name matches we included for every affiliation its relevant sub-affiliations. These sub-affiliations included hospitals with exactly the same name as the university (and *vice versa*) and alternative names of the same affiliation. Using this information, we computed authorship rates of sponsors which were defined as the proportion of registered sponsors that were listed with an author-address on a matched publications.

### 3. Robustness Checks

To ensure that our data is of high quality we conducted three manual checks on 180 publications in our sample that were published in the Journal of the American Medical Association or in the New England Journal of Medicine.

We first compared the reported outcome of the clinical trial in the publication with the primary outcome as described in the registration after reading the registration, the abstracts and if necessary the full text of the matched publications. Of the 180 publications, all but one publication reported on the primary endpoint described in the registration. The single non primary outcome publication described the result of a secondary endpoint.

We subsequently tested our assumption that authorships on primary outcome publications are granted to members of trial management teams. Of 180 publications, 66 publications provided the names of the management team members who were installed in an executive committee or steering committee. 76.7% of all authors on the publications were members of a trial management team or were affiliated with the sponsor. This percentage was 82.9% when only focusing on industry-funded publications.

Third, we checked the quality of the study location data in the registrations by comparing it with the provided information on enrolment efforts in the acknowledgement sections of publications. In only 1.9% of cases was a country listed on a registration, but not mentioned in the acknowledgement of the trial publication.

## Results

### 1. Authorship rates by funding type

The 1,445 registrations listed on average 3.4 enrolment countries in the study location field. In 50.3% of all cases, these enrolment countries did also appear with an author-address on the matched publication (Table 1). The extent to which patient enrolment resulted in authorship on a matched publication was significantly different between funding sources (p<0.001). For government and not-for-profit funded clinical trials, enrolment countries were well represented with authorship rates that surpassed 90%. In contrast, the authorship rate of enrolment countries for industry funded trials was 37.8%. Differences in authorship rates between funding sources were also statistically significant when focusing on a subset of smaller clinical trials that recruited in less than five countries (p<0.001, not shown).

**Table 1:** Number of authors and authorship rates of sponsors and countries.

	Total	Industry	Government	Other not for profit	p-value
**Registration-Publications, n (%)**	1445 (100.0%)	650 (45.0%)	130 (9.0%)	665 (46.0%)	
**Authors, mean (SD)**	9.9 (5.4)	9.8 (5.3)	11.6 (6.3)	9.7 (5.3)	<0.001
**Enrolment countries, mean (SD)**	3.4 (6.2)	6.1 (8.5)	1.2 (0.5)	1.2 (1.2)	<0.001
**Authorship rate of countries** * **, %**	50.3%	39.9%	90.4%	93.7%	<0.001
**Autorship rate of sponsors** ‡ **, %**	80.1%	83.2%	40.0%	84.9%	<0.001

P-values for differences between funding types.

* The authorship rate of countries is defined as the percentage of registered enrolment countries that are listed with an address on a matched publication.

‡ The authorship rate of sponsors is defined as the percentage of registered sponsors that are listed with an affiliation on a matched publication.

Turning to the presence of sponsors on matched publications, in more than 80% of all publications the sponsor became an author on a subsequent publication (Table 1). This percentage was particular high for industry funded clinical trials (83.2%) and other not-for- profit funded clinical trials (84.9%), in comparison to government funded clinical trials (40.0%).

### 2. Authorship rates per country

**Figure 2:**
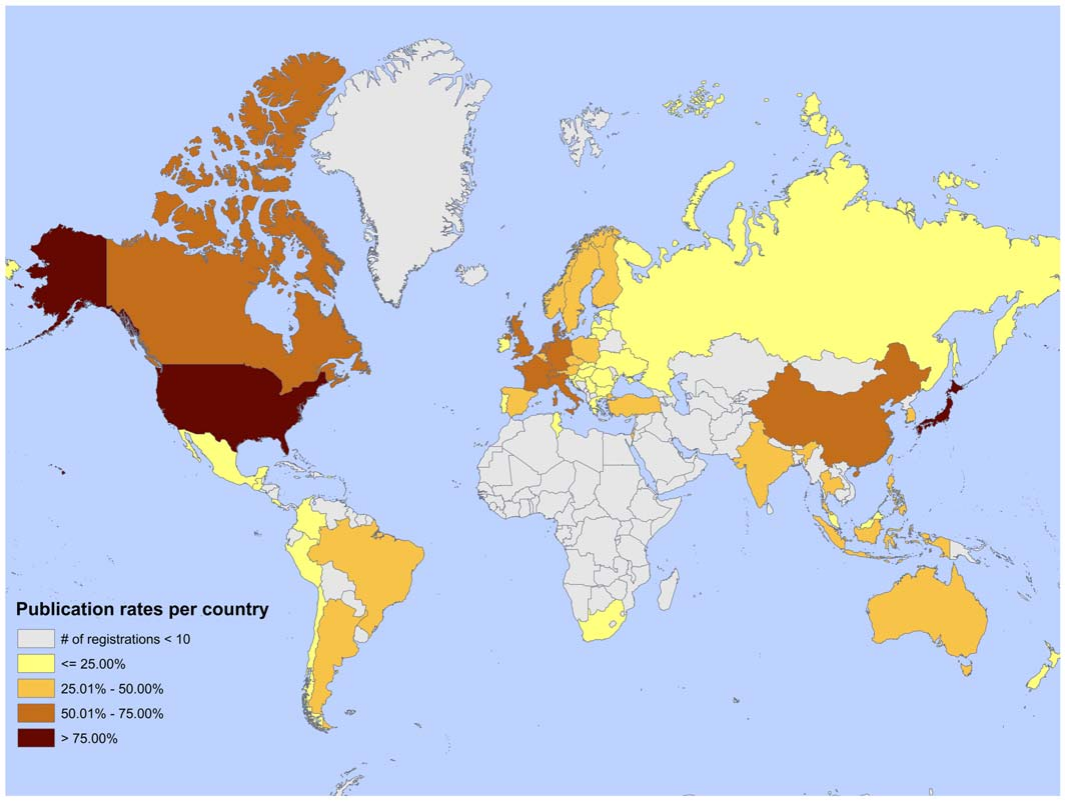
Authorship rates per country for all registrations (n = 1,445).

The extent to which patient enrolment resulted in authorship on a matched publication was unevenly distributed between countries (Figure 2). As shown in Table 1, low authorship rates were mainly visible in industry-funded research and we therefore report country-specific results for this subset of registration-publication pairs in Table 2. Reading from Table 2, the United States was an enrolment country in 399 clinical trials. In 392 of these clinical trials an author-address from the United States appeared on a matched publication, resulting in an authorship rate of 98.2%. In contrast, 92 clinical trials included Mexico as an enrolment country, but an author from Mexico appeared on only 13 matched publications which resulted in an authorship rate of 14.1%. More in general, traditional research locations had the highest authorship rates starting with the United States (98.2%) and followed by Japan (72.0%), Germany (68.0%) and United Kingdom (64.8%). Although enrolment activities were substantial in nontraditional research locations, these countries showed relatively low authorship rates judged for instance by the authorship rates of Poland (27.3%), Czech Republic (23.3%), Argentina (24.1%) and South Africa (22.5%).

**Table 2:** Number of registrations and authorship rates for industry-funded trials per country for all authors and when excluding author-addresses of sponsors.

		Authorship rates	
Country	Number of registrations	All authors	excluding author-addresses of sponsors
United States	399	392 (98.2%)	370 (92.7%)
Germany	194	132 (68.0%)	128 (66.0%)
Canada	171	102 (59.6%)	100 (58.5%)
France	165	95 (57.6%)	93 (56.4%)
Spain	146	57 (39.0%)	57 (39.0%)
United Kingdom	142	92 (64.8%)	86 (60.6%)
Italy	127	54 (42.5%)	53 (41.7%)
Belgium	119	43 (36.1%)	41 (34.5%)
Netherlands	115	47 (40.9%)	46 (40.0%)
Australia	110	40 (36.4%)	39 (35.5%)
Poland	110	30 (27.3%)	30 (27.3%)
Sweden	97	40 (41.2%)	36 (37.1%)
Mexico	92	13 (14.1%)	13 (14.1%)
Denmark	91	43 (47.3%)	39 (42.9%)
Czech Republic	90	21 (23.3%)	21 (23.3%)
Argentina	87	21 (24.1%)	21 (24.1%)
Russia	85	19 (22.4%)	19 (22.4%)
Brazil	84	24 (28.6%)	24 (28.6%)
South Africa	80	18 (22.5%)	18 (22.5%)
Hungary	71	11 (15.5%)	11 (15.5%)
Austria	69	14 (20.3%)	14 (20.3%)
Finland	69	16 (23.2%)	16 (23.2%)
Switzerland	69	25 (36.2%)	18 (26.1%)
Norway	65	11 (16.9%)	11 (16.9%)
Greece	58	7 (12.1%)	7 (12.1%)
China	57	27 (47.4%)	27 (47.4%)
South Korea	54	13 (24.1%)	13 (24.1%)
India	53	16 (30.2%)	16 (30.2%)
Israel	49	8 (16.3%)	8 (16.3%)
Romania	49	8 (16.3%)	8 (16.3%)
Taiwan	47	13 (27.7%)	13 (27.7%)
Puerto Rico	46	8 (17.4%)	8 (17.4%)
Portugal	45	5 (11.1%)	5 (11.1%)
Turkey	43	8 (18.6%)	8 (18.6%)
Slovakia	42	6 (14.3%)	6 (14.3%)
Chile	40	1 (2.5%)	1 (2.5%)
Bulgaria	35	4 (11.4%)	3 (8.6%)
New Zealand	31	7 (22.6%)	7 (22.6%)
Singapore	31	5 (16.1%)	5 (16.1%)
Ireland	29	3 (10.3%)	2 (6.9%)
Malaysia	26	4 (15.4%)	4 (15.4%)
Thailand	26	5 (19.2%)	5 (19.2%)
Japan	25	18 (72.0%)	18 (72.0%)
Ukraine	25	1 (4.0%)	1 (4.0%)
Lithuania	23	3 (13.0%)	3 (13.0%)
Peru	23	3 (13.0%)	3 (13.0%)
Philippines	22	6 (27.3%)	6 (27.3%)
Latvia	21	1 (4.8%)	1 (4.8%)

* Note that a matched publication can contain both an author-address from a sponsor in a particular country and another author-address from a non-sponsor (e.g. university, hospital etc.) in the same country. We only excluded the sponsor related author-addresses in this case.

### 3. Exclusion of author-addresses of sponsors

A reason behind the high authorship rates of traditional research locations might be that pharmaceutical companies are more often located in those countries. This might have biased our results towards countries that host many pharmaceutical companies, because authors from these companies were also often represented on matched publications. We therefore controlled for this in the second set of columns in Table 2, where we assessed authorship rates of enrolment countries, after removing all addresses of authors affiliated to sponsors from the matched publications. This rendered only a minor decrease in the overall authorship rate of countries and did not change the main observation. For instance, the authorship rate of the United Kingdom was 64.8% when considering all authors on matched publications and 60.6% with the exclusion of author-addresses of sponsors. More specifically, the United Kingdom was listed as an enrolment country on 142 registrations and appeared with an author-address on 92 matched publications, which was nearly similar as the 86 matched publications when excluding author-addresses of sponsors. In general, when excluding author-addresses of sponsors, authorship rates remained high for countries that hosted many pharmaceutical companies and for traditional research locations when compared to nontraditional research locations.

## Discussion

This study showed that although clinical trial activities are now executed across the globe, scientific leadership in these trials is disproportionally concentrated in traditional research locations. This geographical decoupling of patient enrolment and clinical trial management is most pronounced in industry funded research. Although we did not empirically investigate the reasons for this phenomenon, we provide three explanations and their implications below.

First, the appointment in trial management teams is dependent on the social structure in which the activities of sponsors and principal investigators are embedded. Authorships of researchers who hold central positions in scientific networks are likely to boost the credibility and dissemination of clinical trial results. It follows that researchers with well-established reputations and affiliations to renowned medical institutes (so-called key opinion leaders) become more likely to be part of management teams than clinical investigators from nontraditional research locations [7]. Over time, these social network structures will be strengthened by the development of trust based relations that facilitate repetition of existing social ties [8], [9]. Given these social network dynamics, the observed geography of scientific leadership becomes performative, as the key roles of researchers in traditional research locations are ‘confirmed’ over and over again at the expense of the roles of researchers in nontraditional research locations.

Second, the structure of clinical trial management might be driven by considerations of efficiency among team members. Despite recent advances in information and communication technologies, face-to-face interaction remains important to carry out the complex tasks associated with scientific research [10], [11]. Accordingly, in selecting clinical trial leaders, pharmaceutical companies might have a preference for researchers that are proximate to them both in geographical terms and on other dimensions, as proximity facilitates efficient communication among team members and decreases coordination costs [12].

Third, from an organizational perspective, relations between clinical investigators in nontraditional research locations and clinical trial management teams might be mediated by a Contract Research Organization (CRO). Pharmaceutical companies increasingly outsource the operational aspects of clinical trials to CROs who negotiate contracts with clinical sites and monitor data production [13], [14]. The use of CROs creates a relatively distant relation between the management team that initiates and designs the trial and the clinical investigators at study sites. Hence, in outsourced clinical trials the role of clinical investigators in other tasks than patient enrolment is frequently modest.

All three arguments point towards distant connections between the researchers that enroll patients and the researchers that produce clinical knowledge for publications. This has implications for the integrity of individual clinical trials and the clinical research enterprise as a whole. With regard to the integrity of individual clinical trials an important implication follows from the increased diversity of patients and their habits in global clinical trials. It is well known that responses to treatment differ considerably between patients according to local diets, drug adherence, body sizes, genetic makeup and the local health care delivery system [1], [5]. Proper interpretation of data therefore necessitates close interactions between those researchers that are in immediate contact with patients and researchers that design trials and interpret clinical trial results. The transfer of context specific knowledge may therefore be best served by increased leadership for researchers from nontraditional research locations. They can create awareness about local specificities and may stimulate debate about the extent to which the findings of clinical trials are generalizable to varying populations across the globe.

Another implication relates to the quality of data that follows from globalized clinical trial conduct. Although it is difficult to make definitive statements here, physicians and researchers from nontraditional countries are often trained in different contexts and are generally less experienced in conducting clinical trials. In addition, they are often not involved in the final knowledge production process and do not always have access to the data they collected [14], [15], [16]. These circumstances may lower their incentive to be accurate in data-collection. Rigorous training of local researchers and increased engagement at leadership level can improve data quality because researchers are made accountable for the final scientific evidence that is produced. Indeed these measures should be taken in addition to strict independent monitoring and regulatory oversight of clinical sites, which is under increasing pressure as indicated by the observation that the FDA inspected only 0.7% of all foreign clinical trial sites in 2008 [17].

A final implication concerns the transparency of global clinical trials. Globalization has made the conduct of clinical trials more decentralized, and it has become difficult to monitor its rapidly changing geography [1]. In this respect, specific worries have been voiced over the relative invisibility of nontraditional research locations in the clinical trial enterprise [5], [6]. Efforts to increase transparency by listing information on enrolment efforts in registrations and publications constitute important steps to change this situation. The recording of enrolment efforts can potentially go beyond the current state to include the names of clinical researchers, the organization that recruit patients and the (expected) number of patients that are recruited per study location. These forms of transparency will make both sponsors and local researchers more accountable for the choices they make and their subsequent performance [18].

Increased transparency about enrolment efforts and the equal inclusion of researchers in clinical trial management is an important step to steer clinical research in a direction where it serves the health needs of communities across the globe. The globalization of clinical trials has raised many ethical concerns including the ethical standards of care that should be provided to patients, obtaining informed consent from illiterate patients and the provision of treatments to patients after the study has ended. It seems in the best interest of patients that researchers from nontraditional research locations have a clear voice in these issues when clinical trials are designed and conducted and when their results are interpreted. This will raise more awareness of the promises and pitfalls of realizing inclusive evidence-based medicine that is to the benefit of patients and researchers across the globe.
